# *Aspergillus flavus* induced oxidative stress and immunosuppressive activity in *Spodoptera litura* as well as safety for mammals

**DOI:** 10.1186/s12866-021-02249-4

**Published:** 2021-06-14

**Authors:** Mandeep Kaur, Pooja Chadha, Sanehdeep Kaur, Amarjeet Kaur

**Affiliations:** 1 Department of Zoology, Guru Nanak Dev University, Amritsar, Punjab India; 2 Departments of Microbiology, Guru Nanak Dev University, Amritsar, Punjab India

**Keywords:** *Spodoptera litura*, *Aspergillus flavus*, Oxidative stress, Lipid peroxidation, Antioxidant enzymes, Haemocytes, Cytotoxicity, Immunosuppressant, Genotoxicity

## Abstract

**Background:**

In the last few decades, considerable attention has been paid to entomopathogenic fungi as biocontrol agents, however little is known about their mode of action and safety. This study aimed to investigate the toxicity of *Aspergillus flavus* in insect *Spodoptera litura* by analyzing the effect of fungal extract on antioxidant and cellular immune defense. In antioxidant defense, the lipid peroxidation (Malondialdehyde content) and antioxidant enzymes activities (Catalase, Ascorbate peroxidase, Superoxide dismutase) were examined. In cellular immune defense, effect of *A. flavus* extract was analyzed on haemocytes using Scanning Electron Microscopy (SEM). Furthermore, mammalian toxicity was analyzed with respect to DNA damage induced in treated rat relative to control by comet assay using different tissues of rat (blood, liver, and kidney).

**Results:**

Ethyl acetate extract of *A. flavus* was administrated to the larvae of *S.litura* using artificial diet method having concentration 1340.84 μg/ml (LC_50_ of fungus). The effect was observed using haemolymph of insect larvae for different time intervals (24, 48, 72 and 96). In particular, Malondialdehyde content and antioxidant enzymes activities were found to be significantly (*p* ≤ 0.05) increased in treated larvae as compared to control. *A. flavus* ethyl acetate extract also exhibit negative impact on haemocytes having major role in cellular immune defense. Various deformities were observed in different haemocytes like cytoplasmic leakage and surface abnormalities etc. Genotoxicity on rat was assessed using different tissues of rat (blood, liver, and kidney) by comet assay. Non-significant effect of *A. flavus* extract was found in all the tissues (blood, liver, and kidney).

**Conclusions:**

Overall the study provides important information regarding the oxidative stress causing potential and immunosuppressant nature of *A. flavus* against *S. litura* and its non toxicity to mammals (rat), mammals (rat), suggesting it an environment friendly pest management agent.

**Supplementary Information:**

The online version contains supplementary material available at 10.1186/s12866-021-02249-4.

## Background

Food security and environment safety are the major concerns in ever expanding human population on the earth planet. Each and every year insect pests cause a severe damage in agricultural field which cost billions of dollars annually to farmers. To reduce the crop damage due to insect pests, the reliance on chemical pesticides has increased. However, irrespective use of chemical pesticides such as Endosulfan, Benzene hexachloride, Aldicarb, and Fenobucarb in agricultural field raised several types of issues include their non-biodegradable nature, development of insecticide resistance and adverse effects on human health as well as environmental concerns [1]. Overall, synthetic pesticides wreak havoc on the environment, threatening biodiversity and human survival [2]. Thus, there is a need to explore alternative ecofriendly strategies for pest’s management which protect and strengthen natural ecosystems rather than contaminate.

Biological control using fungi is one of the most promising technique, due to their unique mechanism of action while infection, low cost, specificity and safety to ecosystem [3, 4]. Entomopathogenic fungi are natural pest controlling agents manifesting immense significance to be used as mycoinsecticides against wide range of insect pests. Among entomopathogenic fungi *Beauveria bassiana* and *Metarhizium anisopliae* have been extensively explored as insecticidal agents and their formulations are also commercially available. Moreover various other fungal spp. viz. *Aspergillus* sp., *Alternaria* sp. and *Nomuraea* sp. have also been disclosed to be entomopathogenic [5–8]. Different *Aspergillus* spp. viz. *A. ochraceus, A. kanagawaensis*, *A. sulphureus, A. flavus* and *A. ochraceus* were found to be pathogenic against several insects such as *Aedes fluviatilis* (Lutz), *Culex quinquefasciatus* (Say), *Anopheles gambiae* (Giles), *Oligonychus coffeae (*Nietner) [9–12]. The insecticidal activity of fungi could be attributed to different secondary metabolites produced by them. Mycotoxins viz. aflatoxins, ochratoxins, fumonisins, zearalenone, have great importance in agriculture for pest management [13]. Various fungal secondary metabolites like avermectins, destruxins, pantherine, ibotenic acid, and tricholomic acid were found to be highly active against insects [14].

Although various studies have been done on the role of fungi as an insect pathogenic agents but many of them failed to address the mode of action. In order to discover the insecticidal potential, the effect on antioxidant and cellular immune defense in insects would be evaluated. Insects possess antioxidant and cellular immune defense system to ward of infection. Antioxidant defense system comprises various antioxidants enzymes which are catalase (CAT), glutathione-S-transferases (GSTs), peroxidase (POX), and superoxide dismutase (SOD). All these play an important role in protecting cells and maintaining homeostasis by removing oxidative stress [15]. Various xenobiotics incite the production of reactive oxygen species (ROS) cause the oxidative stress which ultimately induces oxidative damage, cytotoxicity or immunotoxicity and an increase in insects’ mortality [16]. Cellular immune defense in insects is accomplished through haemocytes. They consists the mixture of cells having different morphological and biological functions and help in providing defense against parasites, pathogens and other foreign bodies enter in the hemocoel [17–20]. Change in number and configuration of haemocytes ultimately affect the immunity and health of insects [21]. So, these parameters are significant while estimating the stress caused by xenobiotics.

On the basis of aforementioned discussion, the study examines the toxicity of ethyl acetate extract of *A. flavus* on insect *Spodoptera litura* (Fabricius), one of the major polyphagous pests, by analyzing the effect on antioxidant and cellular immune defense of insect.

However, if secondary metabolites of fungi are found to deter insects then it would be equitably important to detect whether these metabolites have any mammalian toxicity. As, various chronic diseases have been associated with pesticides exposures, including reproductive or developmental disorders, neurological disorders, cancer etc. Epidemiological studies suggested that occupationally exposed populations to pesticides like pesticide applicators, pesticide manufacturing workers and field workers have developed the high risk of cancer which is due to genomic damage [22–24]. So it is necessary to check genotoxicity of the agent which can be used as pesticide in order to decipher its effects on other non-target species. Various genotoxic markers are chromosomal abbretions (CA), sister chromatid exchange (SCE), micronuclei (MN), comet assay (CO). Comet assay or SCGE is one of the finest techniques for qualitative and quantitative analysis of DNA damage and repair. It was extensively explored in mammal and human cell studies [25–27] and successfully applied on the cells of various animal groups [28]. So in the present study this technique was used to assess the genotoxicity of *A. flavus* on mammals using rat as an animal model to confirm its safety on mammals.

## Results

### Toxicity test of fungus and LC50 value against *S.litura*

The entomopathogenic fungus, *A. flavus* was tested at different concentrations for toxicity against larvae of *S. litura*. The mortality percentages were proportional to extract concentration as shown in Table 1. Different concentrations of the extract caused 16.66–56.66% mortality as compared to 10% in control. The concentrations ranging between 500 and 2000 μg/ml resulted in a significantly higher mortality with respect to control (F = 8.38, *p* ≤ 0.01) (Table 1). The LC50 value of ethyl acetate extract of *A. flavus* as calculated by probit analysis using SPSS software was found to be 1340.84 μg/ml.

**Table 1:** Mortality of *S. litura* larvae fed on diet supplemented with different concentrations of *A. flavus*

Concentrations (μg/ml)	Larval mortality (%) (Mean ± S.E)
Control	10.00 ± 4.47^a^
125	16.66 ± 8.02^ab^
250	26.66 ± 4.21^abc^
500	36.67 ± 6.15^bcd^
1000	43.33 ± 5.24^cd^
2000	56.66 ± 6.14^d^
F value	8.38**

** (*p* ≤ 0.01)

The values are mean ± standard error. Different letters within the column are significantly different (Tukey’s test, p ≤ 0.05) and signify the effect of concentration

### Effect on malondialdehyde (MDA) content and antioxidant enzymes

**Fig. 1:**
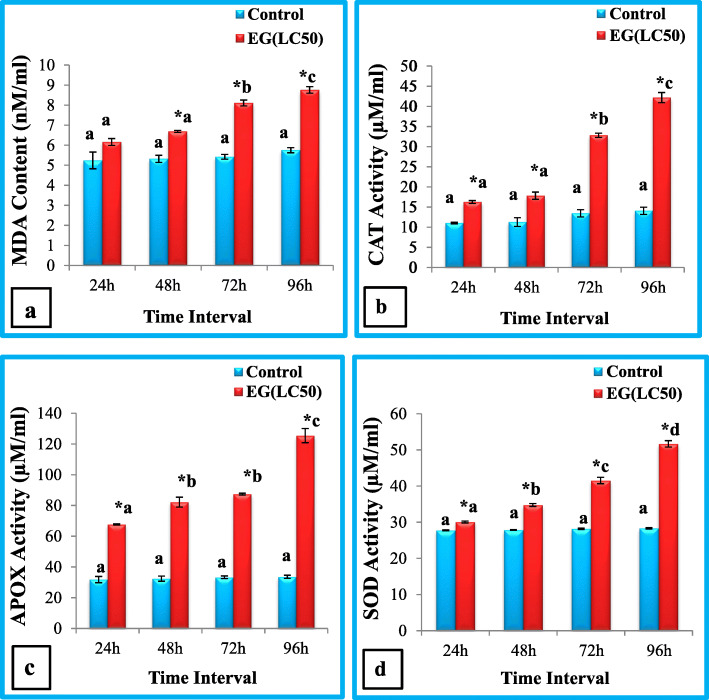
(**a-d**)**:** Malondialdehyde (MDA) content (**a**), Catalase (CAT) activity (**b**), Ascorbate peroxidase (APOX) activity (**c**) and Superoxide dismutase (SOD) activity (**d**) in haemolymph of *S.litura* after treatment with ethyl acetate extract of *A.flavus* for different time intervals. EG = Exposed group. Bars represent mean ± S.E. *Ascribes the significant difference between exposed group and control group (t-test, *p* ≤ 0.05). Different letters **a**, **b**, **c**, **d** are significantly different (Tukey’s test, p ≤ 0.05) and signify the effect of duration

Larvae treated with ethyl acetate extract of *A. flavus* showed hike in level of lipid peroxidation as indicated by MDA content which significantly increased in all treated groups as compared to control in haemolymph of *S. litura* (Student’s t-test) except for 24 h group in which non-significant increase was observed. MDA content was maximum at 96 h (8.76 ± 0.16nmoles/ml) which was significantly higher from control (5.75 ± 0.13nmoles/ml) (t = 14.80, *p* ≤ 0.01). One Way ANOVA reveals the effect of duration was found to be significant (F = 76.14, p ≤ 0.01). Further Tukey’s test reveals the significant difference between 48, 72 and 96 h exposure groups (Fig. 1a).

**Fig. 2:**
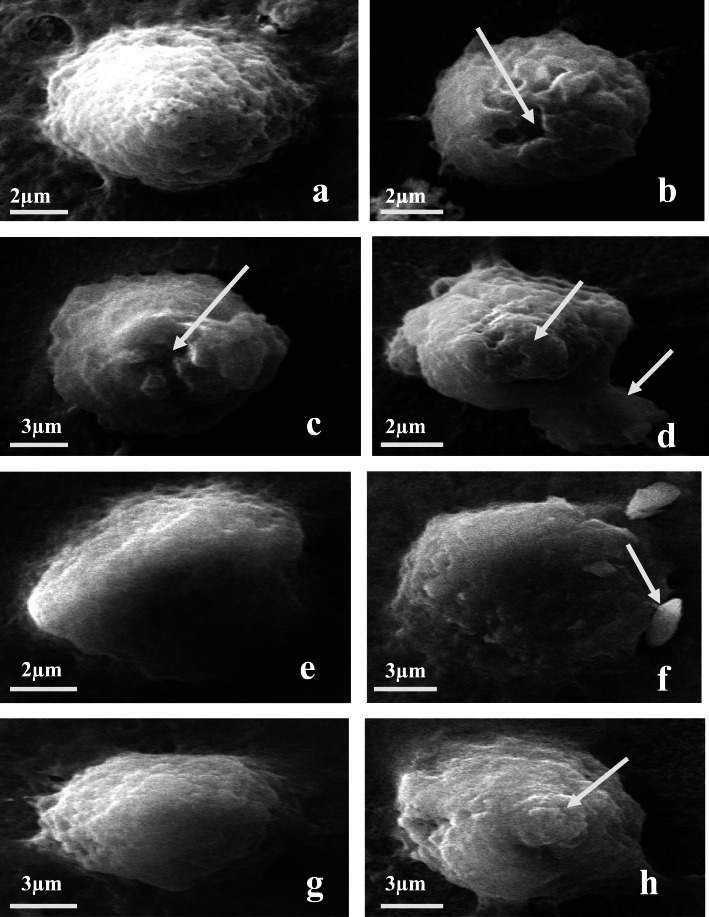
Microphotographs (**a**-**d**) showing Group-1 haemocytes (**a**). Normal haemocytes (Group-1) (b-d). Various deformities observed in haemocytes after treatment with ethyl acetate extract of *A. flavus* (**b**-**c**). Cell perforation (**d**). Surface abnormalities and Cytoplasmic leakage. Microphotographs (**e**-**f**) showing Group-2 haemocytes (**e**). Normal haemocyte; (**f**). Strumae and surface abnormalities in haemocytes after treatment with ethyl acetate extract of *A.flavus.* Microphotographs (**g**-**h**) showing Group-3 haemocytes (**g**). Normal haemocytes (**h**) surface abnormalities in haemocytes after treatment with ethyl acetate extract of *A.flavus*

Statistically significant (t test, *p* ≤ 0.05) increase in activities of all enzymes with respect to control of the exposed *S.litura* larvae was observed. Figure 2b reflected the significant (t test, p ≤ 0.05) hike in catalase (CAT) activity of treated larvae as compared to control. A measured value of CAT was 3 fold higher than control one, for 96 h exposure time group. The difference between all exposure time groups was statistically significant (ANOVA) and significant difference observed between 48 h, 72 h and 96 h groups (Tukey’s test) (Fig. 1b).

An upsurge in Ascorbate peroxidase (APOX) activity was noticed in haemolymph of *S. litura* after treatment with *A.flavus* ethyl acetate extract. The level of enzyme activity was found to be 67.77 ± 0.34, 82.20 ± 3.23, 87.42 ± 0.56, 125.80 ± 4.59μmole/ml following treatment up to 24 h, 48 h, 72 h and 96 h respectively. Highest activity was found in 96 h exposure group where almost 3.74 fold increase was observed in treated groups as compared to control (t = 19.60, *p* ≤ 0.01). Time dependent significant effect was observed (ANOVA) (F = 76.81, p ≤ 0.01) however significant changes were observed between 24 h and 48 h, 72 h and 96 h exposure groups (Tukey’s test) (Fig. 1c).

Superoxide dismutase (SOD) activity also found to be increase in larvae fed with diet amended with ethyl acetate extract of *A. flavus* (Fig. 1d). Significant (t test, *p* ≤ 0.05) rise in SOD activity was occurred in all exposure groups as compared to control however highest activity was obtained at 72 h and 96 h exposure groups where activity increased from 28.19 ± 0.17 μmol/ml (control) to 41.54 ± 0.87 μmol/ml (exposed group) and 28.33 ± 0.18 μmol/ml (control) to 51.68 ± 0.88 μmol/ml (exposed group) respectively. With increase in time duration the enzyme activity was significantly increased (ANOVA) (F = 202.57, *p* ≤ 0.01). Significant difference observed between 48 h, 72 h and 96 h exposure groups (Tukey’s test) (Fig. 1d).

### Effect on haemocytes

The scanning electron microscopy studies revealed that the haemocytes of *S.litura* were changed very apparently after treatment with *A.flavus* ethyl acetate extract (Fig. 2). After 96 h various morphological deformities were observed in different haemocytes. As compared to normal haemocytes (Group-1) treated ones showed cell perforation and cytoplasmic leakage (Fig. 2a-d). Similarly, normal haemocytes of Group-2 not shown any deformity but treated ones showed strumae and surface abnormalities (Fig. 2e-f). Group − 3 haemocytes showed surface abnormalities after treatment with *A.flavus* (2 g-h). Overall SEM studies revealed that morphology of haemocytes was highly disrupted after treatment with ethyl acetate extract of *A.flavus* for 96 h which might be leads to cytotoxicity.

**Fig. 3:**
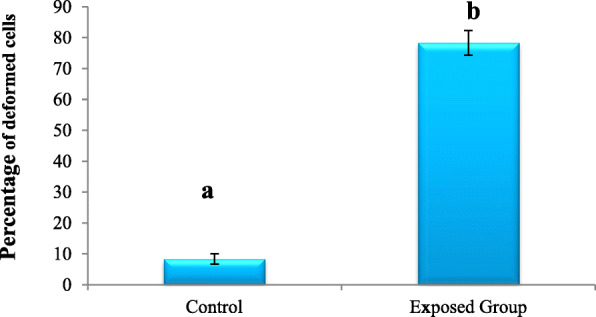
The percentage of cells showing various deformities

Relative to the control, the percentage of haemocytes with various deformities were found to be significantly increased in treated larvae due to the toxic effects of fungal extract. After 96 h of feeding, the percentage of cells having various deformities were 78.33% as compared to 8.33% in control (Fig. 3).

### Mammalian toxicity

**Fig. 4:**
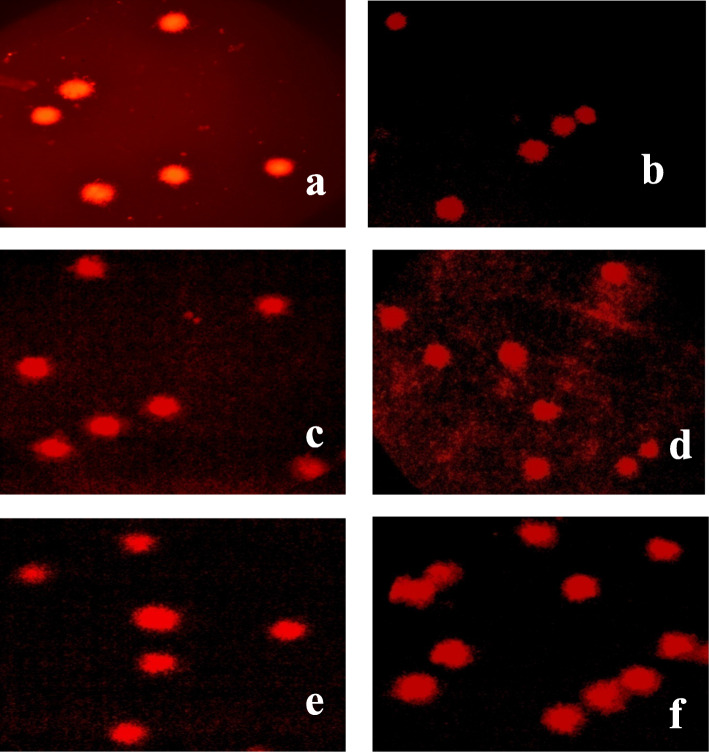
Photomicrographs showing DNA extracted from (**a**, **b**) rat blood cells (**a**) Control; (**b**) After treatment with *A.flavus* ethyl acetate extract (**c**, **d**) rat kidney cells (**a**) Control; (**b**) After treatment with *A.flavus* ethyl acetate extract (**e**, **f**) rat liver cells (**a**) Control; (**b**) After treatment with *A.flavus* ethyl acetate extract

Comet assay was conducted to assess genotoxicity on rat using parameters, Tail length, % Tail DNA, Tail Moment and Olive Tail Moment. The obtained data in Tables 2, 3, 4 showed that there were no significant differences in all the parameters after 24 and 96 h of rat’s administration with *A.flavus* extract at dose level of 100 mg/kg body weight and 200 mg/kg body weight relative to control (ANOVA). In case of rat blood non-significant increase was observed for all the parameters except in case of tail length of 96 h group where significant increase was observed from 14.78 ± 0.13 (control) to 15.51 ± 0.11 (200 mg/kg b.wt). In all other groups non-significant increase was observed after treatment with different concentrations of *A. flavus* (Table 2). Similarly in rat liver non-significant effect was obtained for all the parameters after treatment with both concentrations ethyl acetate extract of *A. flavus* (ANOVA) (Table 3). In rat kidney all parameters showed non-significant increase except tail length of 24 h group and %tail DNA of 96 h group where significant increase was observed (Table 4). Effect of duration was also found to be non-significant in all the tissues as revealed by student’s t-test. Overall non-significant effect was observed. Photomicrographs showing DNA damage in different tissues of rat after treatment with *A. flavus* fungal extracts are shown in Fig. 4.

**Table 2:** Effect of different concentrations of ethyl acetate extract of *A. flavus* on different parameters of comet assay in rat blood

**Tail length (**μm**)**	**24**	**96**	**t value**	**% Tail DNA**	**24**	**96**	**t value**
**Control**	14.57 ± 0.18^a^	14.78 ± 0.13^a^	1.48	**Control**	4.73 ± 0.41^a^	5.13 ± 0.09^a^	0.58
**100 mg/kg**	14.84 ± 0.21^a^	14.99 ± 0.10^a^	0.78	**100 mg/kg**	4.84 ± 0.02^a^	5.19 ± 0.23^a^	0.54
**200 mg/kg**	15.14 ± 0.04^a^	15.51 ± 0.11^b^	1.40	**200 mg/kg**	4.99 ± 0.45^a^	5.52 ± 0.18^a^	1.31
**F value**	2.932	14.738**		**F value**	1.130	1.378	
**Tail Moment (AU)**	**24**	**96**	**t value**	**Olive Tail Moment (AU)**	**24**	**96**	**t value**
**Control**	1.54 ± 0.27^a^	1.64 ± 0.32^a^	0.48	**Control**	2.01 ± 0.07^a^	2.14 ± 0.15^a^	0.75
**100 mg/kg**	1.65 ± 0.11^a^	1.44 ± 0.11^a^	1.38	**100 mg/kg**	2.11 ± 0.05^a^	2.45 ± 0.06^a^	19.27
**200 mg/kg**	2.02 ± 0.10^a^	2.33 ± 0.15^a^	0.66	**200 mg/kg**	2.25 ± 0.01^a^	2.78 ± 0.29^a^	3.71
**F value**	0.248	0.062		**F value**	4.527	2.72	

** (*p* ≤ 0.01), The values represented as mean ± standard error. Different letters a, b within the columns are significantly different (Tukey’s test, *p* ≤ 0.05) and signify the effect of concentration

**Table 3:** Effect of different concentrations of ethyl acetate extract of *A. flavus* on different parameters of comet assay in rat liver

**Tail Length (μm)**	**24**	**96**	**t value**	**% Tail DNA**	**24**	**96**	**t value**
**Control**	12.44 ± 0.15^a^	12.69 ± 0.23^a^	5.55	**Control**	4.07 ± 0.29^a^	4.22 ± 0.23^a^	2.08
**100 mg/kg**	12.67 ± 0.20^a^	12.84 ± 0.33^a^	9.33	**100 mg/kg**	4.62 ± 0.22^a^	4.78 ± 0.11^a^	21.56
**200 mg/kg**	13.03 ± 0.27^a^	12.92 ± 0.36^a^	1.40	**200 mg/kg**	4.79 ± 0.25^a^	4.64 ± 0.21^a^	3.46
**F value**	1.852	0.182		**F value**	2.124	2.314	
**Tail Moment (AU)**	**24**	**96**	**t value**	**Olive Tail Moment (AU)**	**24**	**96**	**t value**
**Control**	1.16 ± 0.15^a^	1.34 ± 0.14^a^	0.84	**Control**	1.46 ± 0.19^a^	1.44 ± 0.18^a^	1.82
**100 mg/kg**	1.47 ± 0.13^a^	1.44 ± 0.21^a^	0.12	**100 mg/kg**	1.52 ± 0.07^a^	1.57 ± 0.06^a^	17.50
**200 mg/kg**	1.31 ± 0.10^a^	1.70 ± 0.15^a^	2.07	**200 mg/kg**	1.51 ± 0.20^a^	1.85 ± 0.03^a^	2.14
**F value**	1.423	1.133		**F value**	0.530	3.277	

The values represented as mean ± standard error. Different letters a, b within the columns are significantly different (Tukey’s test, *p* ≤ 0.05) and signify the effect of concentration

**Table 4:** Effect of different concentrations of ethyl acetate extract of *A. flavus* on different parameters of comet assay in rat kidney

**Tail** **Length** **(μm)**	**24**	**96**	**t value**	**% Tail DNA**	**24**	**96**	**t value**
**Control**	14.95 ± 0.22^a^	15.16 ± 0.39^a^	0.34	**Control**	4.74 ± 0.14^a^	4.53 ± 0.04^a^	1.39
**100 mg/kg**	15.18 ± 0.37^ab^	15.21 ± 0.15^a^	0.78	**100 mg/kg**	4.96 ± 0.09^a^	5.07 ± 0.16^b^	0.54
**200 mg/kg**	15.38 ± 0.02^b^	15.57 ± 0.04^a^	0.43	**200 mg/kg**	5.05 ± 0.30^a^	5.06 ± 0.02^b^	0.36
**F value**	0.717	0.842		**F value**	0.640	9.92*	
**Tail Moment (AU)**	**24**	**96**	**t value**	**Olive Tail Moment (AU)**	**24**	**96**	**t value**
**Control**	1.70 ± 0.15^a^	1.72 ± 0.16^a^	2.44	**Control**	1.93 ± 0.32^a^	2.01 ± 0.31^a^	0.12
**100 mg/kg**	1.92 ± 0.05^a^	1.91 ± 0.31^a^	1.38	**100 mg/kg**	1.99 ± 0.01^a^	1.96 ± 0.35^a^	19.72
**200 mg/kg**	2.12 ± 0.13^a^	2.31 ± 0.09^a^	1.90	**200 mg/kg**	2.10 ± 0.15^a^	2.17 ± 0.17^a^	1.98
**F value**	2.970	0.986		**F value**	0.189	0.141	

* (*p* ≤ 0.05). The values represented as mean ± standard error. Different letters a, b within the columns are significantly different (Tukey’s test, *p* ≤ 0.05) and signify the effect of concentration

## Discussion

In the last few decades, entomopathogenic fungi have attracted considerable attention as biocontrol agents in sustainable agriculture. They are known to have several advantages as compared to synthetic pesticides. To date, several studies have indicated the fungal agents as insect pathogens [5, 6, 29, 30]. So, in the present study secondary metabolites of *A. flavus* were extracted using ethyl acetate and tested for their toxicity on insect, *Spodoptera litura*. Ethyl acetate extract was found to exhibit negative impact on larval survival. Previously various species of *Aspergillus* are found to be entomopathogenic like *A.flavus, A.oryzae*, *A.tamarii* and *A.versicolor*, *A.parasititus* etc. [5, 6, 31, 32]. Two strains of *A. flavus* named as *A. flavus* NRRL and *A. flavus* AF36 are commercially available as biocontrol agents [33, 34]. Their active ingredients are being used in various pesticides which also help in reducing aflatoxin contamination in crops.

To decipher the mode of action the effect on antioxidant and cellular immune response was studied. Insects have evolved multiple defense mechanisms including antioxidant and cellular immune defense, to respond to pathogens. Antioxidant defense systems in insects include various antioxidant enzymes viz. Catalase (CAT), Ascorbate peroxidase (APOX), Superoxide dismutase (SOD) and Glutathione-S-Transferase (GST). All work co-coordinately to maintain the state of dynamic equilibrium in organism, keeping ROS low level to prevent the cells from damage [35, 36]. Increase in malondialdehyde *(*MDA) content and antioxidant enzymes activity is an important indicator of oxidative stress. MDA is the product formed during lipid peroxidation (LP) which cause DNA damage and cell death [37]. So, in this study, the MDA content and activities of antioxidant enzymes were determined in *Spodoptera litura* larvae at different times, after treatment with ethyl acetate extract of *A.flavus,* in order to speculate *A.flavus* induced oxidative stress and effect on antioxidant defense*.* Results showed significant increase in MDA level in response to fungal extract as compared to control. At 96 h value increased 1.5 times as compared to control. This finding is in corroboration with the finding of Karthi et al. [12] revealing the toxic effects of *Aspergillus flavus* spores on *S.litura*.

The activities of antioxidant enzymes are also found to be significantly increased after exposure to *A.flavus* extract, showing influence of fungus on antioxidant defense of insect. CAT and APOX activities increased 3.00 and 3.74 fold respectively, however, SOD activity was found to be increased 1.82 fold as compared to control, at 96 h, in haemolymph of *S. litura.* This might be due to activation of host response after toxicity induced by *A.flavus*, in which enzymes activities were remarkably accelerated to metabolize the ROS, reaching the maximum value at 96 h. Similar increase in antioxidant enzymes activities [superoxide dismutase (SOD), catalase (CAT), peroxidases (POX)] was observed by Karthi et al. [12] under the influence of *A. flavus* spores. Scarce reports recorded the effect of fungal agents on protective enzymes activities. Ding et al. [38] observed the increase in protective enzymes activity in *Xylotrechus rusticus* (Linnaeus) under the influence of *B. bassiana. M. anisopliae* also found to alter the antioxidant and detoxifying enzymes activities in *Periplaneta americana* (Linnaeus) and *Locusta migratoria* (Linnaeus) [39, 40]. Chaurasia et al. [41] observed variable activity of antioxidant enzymes in *P. americana* under the influence of entomopathogenic fungus *Hirsutella thompsonii*. However alteration in enzymes activities due to different chemicals and other stress factors in insects has been reported by many studies [42–46].

Cellular response in insect immune system acts as an important barrier to the infection process [47, 48]. Haemocytes types and their specific responses while insect–pathogen interaction act as a good indicators of insect defense reactions [49, 50]. There are different types of haemocytes which have been morphologically and functionally characterized in various insects [51–53]. Multifunctional role of haemocytes are phagocytosis, encapsulation, cell agglutination, detoxification etc. Change in number and configuration was observed in haemocytes under different stresses which ultimately affect the health of insects. Consequently these cells have been used to ascertain the cytogenetic damage by toxic chemicals [21, 54].

So, in our investigation effect on cellular immune response was also judged by analyzing effect on haemocytes by scanning electron microscopy. SEM results showed various cellular deformities in different haemocytes like cell perforation, cytoplasmic leakage, strumae and surface abnormalities after treatment with ethyl acetate extract of *A.flavus* as compared to control. The results are in corroboration with study of Fan et al. [55] which observed the cell perforation and rupturing with cytoplasmic leakage in haemocytes of *Bombyx mori* (Linnaeus) after treatment with destruxin A. There are very few studies which reveal the abnormalities of insect haemocytes using SEM, however technique has been used by various researchers to observe and characterize the different types of haemocytes in insects [17, 56] and to observe spores accumulation in insect’s body after fungal infection [5, 6, 57]. Recently Duan et al. [58] observed the infection of *B. bassiana* to *Leptinotarsa decemlineata* (Say) via scanning electron microscopy. However the morphological changes observed in present study were demonstrated by various workers due to entomopathogenic fungi and insecticides under light microscopy [59–61].

Mammalian toxicity of *A.flavus* was also carried out by assessing DNA damage in blood, liver and kidney of rat. Non-significant effect was observed in all the tissues of fungal extracts treated rats as compared to control. Similarly negligible toxicity of azadirachtin, a neem biopesticides was earlier reported in rats [62] and human [63]. There are few other botanical extracts which were also checked for their toxicity on rat such as *Cassia senna, Caesalpinia gilliesii, Thespesia populnea, Chrysanthemum frutescens, Euonymus japonicus, Bauhinia purpurea*, and *Cassia fistula* extracts [64], *Dichaetanthera africana* extract [65]. Toxicity of fungal extracts on mammals was not checked previously however, Sprando et al. [66] reported the safety of bacterial species *Paenibacillus Alvei* to rat.

Moreover, the insecticidal activity of the fungal extract is attributed mainly to various secondary metabolites found in it. Our previous study reported the presence of various phenolic compounds viz. gallic acid, caffeic acid, quercetin and kaempferol by UHPLC in *A. flavus* extract. So different results observed of *A.flavus* in insects and rat may be attributed to the presence of these phenolics. Phenolic compounds act as antioxidants in mammals [67, 68] and pro-oxidants in insects [67, 69–71] which oxidized after consumption by insects and the reaction products formed are responsible for causing oxidative stress [69]. The other thing regarding its safety and non toxicity on mammals is that it’s a non-aflatoxigenic *Aspergillus flavus*, the strain does not produce aflatoxin as proved in our previous study by LC-MS [29]. Previously various non-aflatoxin fungal strains of *A. flavus* are being used to prevent aflatoxin contamination in crops and to control pests [33, 34]. So, on the basis of mammalian toxicity and aflatoxins detection studies, the fungus could be considered safe and can be used as a biocontrol agent by further field trials.

## Conclusion

Overall, the study highlights the adverse effect of *A. flavus* on the physiology of *S.litura* which might be due to the negative impact on antioxidant and cellular immune defense of an insect. The study helps to identify the insect defenses that could be manipulated to accelerate host death in biological control scenario. The study also showed its safety for mammals as it showed negligible toxicity on rat, suggesting it to be used as bio-pesticide after further field studies.

## Methods

### Insects rearing

*Spodoptera litura* (Lepidoptera) eggs were obtained from the cauliflower fields around Amritsar (India). After hatching of eggs larvae were fed on castor leaf. Subsequent generations of culture were maintained in laboratory at 25 ± 2 °C temperature, 65 ± 5% relative humidity and 12:12 (D: L) photoperiod [72].

### Fungal culture isolation, production and identification

Fungus was isolated from the surface of dead insect (*Spodoptera litura*) and named as IL (insect larva) fungus. The cause of death was due to natural mould formation. Mycelium fragments were picked directly from the surface of dead insect and point inoculated on Potato Dextrose Agar (PDA) plates supplemented with ampicillin (200 mg/ml). Plates were incubated at 30 °C. After 2–3 days of incubation mycelium regeneration was transferred to new PDA plates for purification purpose. Purified culture was maintained on PDA and stored at 4 °C [29].

The production was carried out in 50 ml malt extract (malt extract = 20 g/l, dextrose = 20 g/l,peptone = 1 g/l, pH = 5.5) broth in 250 ml Erlenmeyer flask by inoculating one plug (1 cm square) taken from the periphery of an actively growing culture. The flasks were incubated at 30 °C and 250 rpm for 10 days. After 10 days extraction was carried out twice using ethyl acetate at 120 rpm and 40 °C. The extracts were concentrated by using rotavapor and dissolved in 1 ml DMSO and stored at 4 °C.

The IL fungus was identified as *Aspergillus flavus* on morphological (Fig. S1: Morphology of *A.flavus* showing, Hyphae and Conidiophore under SEM) and molecular basis as indicated in our previous study [29] by using ITS1 and ITS4 primer to amplify ITS1–5.8S- rDNA- ITS2 region. Amplified ITS region was Purified and sequenced at first base sequencing (Malaysia). The sequence similarity was matched with other available databases retrieved from NCBI using BLAST [73]. Phylogenetic Analysis was done using sequences obtained after blast which were aligned using ClustalW program. MEGA 7 software was used to construct a phylogeny tree. Evolutionary history was inferred by Neighbor-joining method and Kimura-2-parameter model [29]. The sequence obtained was submitted to GeneBank under the accession number MF680839 [29] and the final name of fungus is *Aspergillus flavus isolate IL* (MF680839) [29].

### Toxicity test of fungus and LC50 value against *S.litura*

Toxicity of fungus was tested by checking mortality rate. For this different concentrations (125, 250, 500, 1000 and 2000 μg/ml) of fungal extract were made in 0.5% DMSO and added in artificial diet. The Second instar larvae (6 days old) were reared on fungal extract amended diets as well as with control diet (0.5% DMSO) at controlled temperature 25 ± 2 °C and relative humidity 70 ± 5% conditions. The experiment was replicated six times with five larvae per replication. Each larva was put in separate container (4 × 6 cm) and the diet was changed daily till pupation. Dead larvae were checked daily till pupation. The total numbers of dead larvae were counted. The toxic effect of fungal extract on *S.litura* was calculated using the probit analysis LC_50_ (lethal concentration) determination method.

### Effect on malondialdehyde (MDA) content and antioxidant enzymes activity

To evaluate the effect of fungal extracts on lipid peroxidation and antioxidant enzymes, the third instar larvae (12 days old) were fed with fungal extracts supplemented diet having concentration 1340.84 μg/ml (LC_50_ of fungus). The MDA content and enzymes activities [Superoxide dismutase (SOD), catalase (CAT), Ascorbate peroxidase (APOX)] were analyzed in haemolymph of third instar (12 days) larvae.

Larvae were divided into two groups, treatment and control. Treatment group was treated with LC_50_ of fungus at controlled temperature 25 ± 2 °C and relative humidity 65 ± 5%. The second group was treated with control diet (0.5% DMSO) at same conditions of temperature and relative humidity. The effect of fungal extract has been recorded after different time intervals (24 h, 48 h, 72 h and 96 h) in lipid peroxidation and enzyme activities. The experiment was replicated three times. For each treatment and control there are 10 larvae per replication were taken.

### Tissue collection

Haemolymph was collected by cutting proleg with microscissor from 10 different larvae fed with same concentration and then it was pooled. Pooled haemolymph (10%) was mixed with PBS (Phosphate Buffer Saline pH 7.0) containing 0.01%phenylthiourea and centrifuged for 20 min at 10000 g, 4 °C and supernatant obtained was used for enzyme activities studies.

The extraction procedure was same for lipid peroxidation and all enzymes.

### Malondialdehyde (MDA) content

MDA content was measured according to Jain and Levine [74] with slight modifications. MDA content as an indicator of lipid peroxidation was determined after incubation of 0.5 ml of sample (supernatant) at 95 °C with Trichloroacetic acid (TCA) (20% w/v), Thiobarbituric acid (TBA) (1% w/v). Absorbance was taken at 532 nm against the blank. MDA content was expressed as nanomole/ml by using 1.56 × 10^5^ M^− 1^ cm^− 1^ extinction coefficient.

### Catalase (CAT) activity

Enzyme activity was estimated according to methodology given by Aebi [75] with slight modifications. 0.1 ml of supernatant was added into 2.9 ml of H_2_O_2_ in a cuvette. Decrease in absorbance was read at 240 nm for 5 min at 1 min interval (25 °C). The enzyme activity was expressed as μM/ml (haemolymph).

### Ascorbate peroxidase (APOX) activity

The enzyme activity was calculated according to methodology given by Asada [76] with slight modifications. 0.1 ml of sample, 0.6 ml extraction buffer and 0.125 ml of 0.3%H_2_O_2_ were taken in cuvette. The decrease in absorbance was recorded at 290 nm for 5 min at 30s interval (25 °C). The enzyme activity was expressed as μM/ml (haemolymph).

### Superoxide dismutase (SOD) activity

The enzyme activity was calculated according to methodology given by Kono [77] with slight modifications. 0.05 ml sample, 1.5 ml sodium carbonate buffer, 0.5 ml NBT (Nitroblue tetrazolium), 0.1 ml TritonX-100, 0.1 ml hydroxylamine hydrochloride were taken in cuvette and increase in absorbance was recorded at 540 nm. The enzyme activity was expressed as μM/ml (haemolymph).

### Effect on haemocytes

Haemolymph was collected and pooled from 10 larvae fed with same concentration. Effect on haemocytes was studied by scanning electron microscopy (SEM).

### Scanning electron microscopy (SEM)

SEM was done according to methodology of Wang et al. [78] with slight modifications. Haemolymph was bled on termanox discs after cutting proleg of larvae. It was allowed to dry and fixed with 2.5% glutaraldehyde in 0.1 M cacodylate buffer (pH 7.2) for two hours. After this sequential dehydration was done by using graded series of ethanol i.e. 25% followed by 50, 70, 90% and at the end with absolute (100%) alcohol. Then discs were placed in dry chamber for proper drying. At the end silver coating was done by mounting samples on aluminium stubs and haemocytes were observed under SEM at magnification of 10.00KX operated at 10KV after 96 h of treatment with fungal extracts.

### Mammalian toxicity study

Sexually mature male wistar albino rats having weight 120 ± 20 g were used in study. Animals were reared on commercial pellet diet and water adds libitum and housed in cages at particular temperature (25 ± 2 °C) and humidity conditions (50–60%). The rats were brought from private source. All experiments were performed according to guidelines provided of Institutional Animal Ethics Committee (IAEC) of Guru Nanak Dev University, Amritsar, Punjab (India). The application for permission for animal experiments was submitted to the CPCSEA, New Delhi and after approval of Institutional Animal Ethics Committee (IAEC) got Registration number: 226/PO/Re/S/2000/CPCSEA. The animals were acclimatized 5 days before experiments. Two concentrations 100 mg/kg b.wt and 200 mg/kg b.wt of fungal extracts were selected for experiments and effects were studied after 24 h and 96 h of exposure. The *A.flavus* fungal extract dissolved in 0.5%DMSO was injected intraperitoneally to the rat. The experiment was replicated thrice and DNA damage was assessed by comet assay according to methodology given by Ahuja and Saran [79] in different tissues viz. blood, liver and kidney. Rats were sacrificed using gas inhalation method. Blood samples (1 ml) were taken directly from heart and used as such after adding anticoagulant however two tissues i.e. liver, kidney were homogenized in PBS and centrifuged at 10,000 g for 10 min. Cell suspension was taken and used for DNA damage study.

### Statistical analysis

To study the effect of duration one way analysis of variance (ANOVA) with Tukey’s test was performed and to study the effect of treatment student’s t-test was applied.

## Supplementary Information

**Additional file 1: Figure S1.** Morphology of *A. flavus*: Hyphae and Conidiophore under SEM.

## Data Availability

All data generated or analyzed during this study are included in this article and its additional files.

## Abbreviations

CAT: Catalase
APOX: Ascorbate peroxidase
SOD: Superoxide dismutase
GST: Glutathione- S-Transferase
