# Supplementary material for: Aspergillus flavus induced oxidative stress and immunosuppressive activity in Spodoptera litura as well as safety for mammals
Source: BMC Microbiol. 2021 Jun 14;21:180. doi: 10.1186/s12866-021-02249-4 (PMC8204525; doi:10.1186/s12866-021-02249-4)
Supplement: Supplementary file 1 — Additional file 1: Figure S1. Morphology of A. flavus: Hyphae and Conidiophore under SEM. [file 12866_2021_2249_MOESM1_ESM.docx]

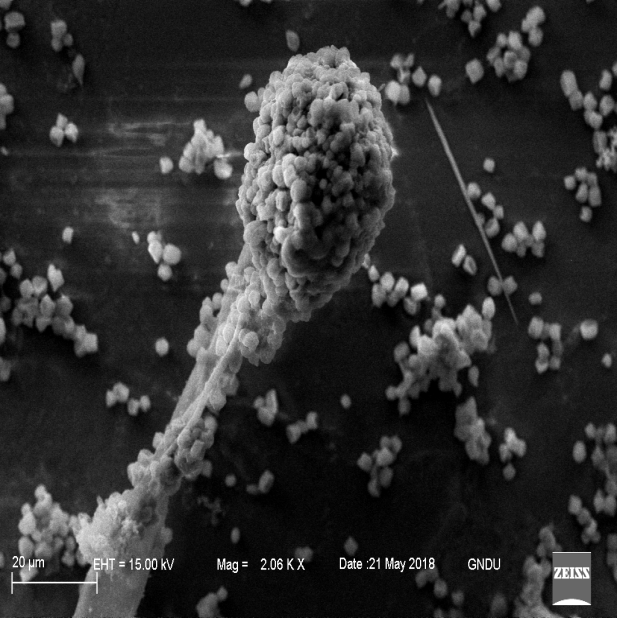


**Fig. S1:** Morphology of *A. flavus*: Hyphae and Conidiophore under SEM.

**Sequence of *A. flavus* (IL):**

**ITS-1 (527BP)**

GATTTACTGTACCTTAGTTGCTTCGGCGGGCCCGCCATTCGTGGCCGCCGGGGGCTCTCAGCCCCGGGCCCGCGCCCGCCGGAGACACCACGAACTCTGTCTGATCTAGTGAAGTCTGAGTTGATTGTATCGCAATCAGTTAAAACTTTCAACAATGGATCTCTTGGTTCCGGCATCGATGAAGAACGCAGCGAAATGCGATAACTAGTGTGAATTGCAGAATTCCGTGAATCATCGAGTCTTTGAACGCACATTGCGCCCCCTGGTATTCCGGGGGGCATGCCTGTCCGAGCGTCATTGCTGCCCATCAAGCACGGCTTGTGTGTTGGGTCGTCGTCCCCTCTCCGGGGGGGACGGGCCCCAAAGGCAGCGGCGGCACCGCGTCCGATCCTCGAGCGTATGGGGCTTTGTCACCCGCTCTGTAGGCCCGGCCGGCGCTTGCCGAACGCAAATCAATCTTTTTCCAGGTTGACCTCGGATCAGGTAGGGATACCCGCTGAACTTAAGCATATCAATAACCGGAGAGA
